# Surgical exposure of an impacted maxillary canine and increasing a band of keratinized gingiva

**DOI:** 10.4103/0972-124X.60232

**Published:** 2009

**Authors:** R. Vijayalakshmi, T. Ramakrishnan, S. Nisanth

**Affiliations:** *Department of Periodontics, Meenakshi Ammal Dental College and Hospital, Chennai - 600 095, India*; 1 *Department of Meenakshi Ammal Dental College and Hospital, Chennai - 600 095, India*; 2 *Department of Meenakshi Ammal Dental College and Hospital, Chennai - 600 095, India*

**Keywords:** Buccal eruption, impacted canine, periodontal surgery

## Abstract

An adequate amount of keratinized gingival tissue that is under proper plaque control, is a fundamental requirement for periodontal health. When the teeth erupt uneventfully in the center of the alveolar ridge, an adequate amount of keratinized tissue will surround the erupted permanent tooth. Labially or buccally erupting teeth show reduced dimensions of the gingiva as abnormal eruption of permanent teeth restricts or eliminates the keratinized tissue between the erupting cusp and the deciduous tooth. A lack of attached gingiva poses a potential risk for gingival recession in labially or buccally erupted teeth due to the possibility of accumulation of plaque and/or traumatic tooth-brushing during subsequent orthodontic treatment. A good understanding between the orthodontist and periodontist along with proper management of periodontal tissues, can prevent these problems. Various surgical techniques can be employed to uncover impacted teeth. This paper discusses the validity of utilizing periodontal surgery to increase a band of keratinized tissue in a case of an impacted canine erupting from the alveolar mucosa.

## INTRODUCTION

The Periodontist is often called on by the Orthodontist for the exposure of an impacted tooth, which is essential for successful orthodontic treatment. The maxillary and mandibular third molars are the most commonly impacted teeth due to their long development time.[1] The maxillary cuspid is the second most frequently impacted tooth (2%).[2] The cuspids are generally one of the last teeth to erupt into the arch and are adversely affected by:[3]

1. The loss of space
2. Overretained deciduous teeth
3. Deflection (facially or palatally) of the lateral incisor

Various treatment modalities have been proposed to avoid the complications associated with impacted canines.[4] These complications include:

1. Internal or external resorption
2. Infection associated with partial eruption
3. Loss of arch length
4. Resorption of the roots of lateral incisors

Also, the position of a tooth erupting through the alveolar process in mixed dentition and its ultimate position in relation to the buccolingual dimension of the alveolar process, can have a profound outcome on the amount of attached gingiva around the tooth.

An adequate amount of keratinized gingival tissue that is under proper plaque control, is a fundamental requirement for periodontal health.[5] When teeth erupt uneventfully in the center of the alveolar ridge, an adequate amount of keratinized tissue will surround the erupted permanent tooth. Labially or buccally erupting teeth show reduced dimensions of gingiva as abnormal eruption of permanent teeth restricts or eliminates the keratinized tissue between the erupting cusp and the deciduous tooth.[6] A lack of attached gingiva poses a potential risk for gingival recession in labially or buccally erupted teeth due to the possibility of accumulation of plaque and/or traumatic tooth-brushing during subsequent orthodontic treatment.[7] A good understanding between the orthodontist and the periodontist along with proper management of periodontal tissues can prevent these problems.

Historically, a number of methods have been described for exposure of impacted teeth:

- Celluloid crown
- Pack the wound area to maintain exposure
- Recommended gutta-percha packing
- Pins
- Orthodontic bands
- Wire ligature

The three most significant advances historically for exposure are:

1. Palatal flap for exposure
2. Direct-bonding brackets
3. Soft tissue management

The palatal flap provided access and visibility. Direct bonding reduced morbidity by minimizing wound size and reduced tissue overgrowth and additional surgeries by having a bracket placed at the time of exposure.

Soft tissue management maintained and permitted an increase in keratinized gingiva, eliminating needless secondary surgery to treat mucogingival problems and prevent recession.[8]

Localization and determination of a tooth's exact position is the foremost step in surgical exposure of an impacted tooth. This can often be done by palpation in labial impactions. However, the use of periapical radiographs and occlusal radiographs plays a major role in palatal and middle alveolar impactions. Use of the buccal object rule is helpful in determining the location of impacted teeth.[9]

Various surgical techniques can be employed to uncover impacted teeth. The vertical location of the permanent tooth position to deciduous tooth and the amount of gingiva available, will determine the selection of the appropriate technique. The goal of these mucogingival interceptive surgeries is to prevent the ectopic permanent tooth from developing periodontal lesions in its most incipient stage.[5] In this paper, a case report is presented to discuss the validity of utilizing periodontal surgery to increase a band of keratinized tissue in a case of an impacted canine erupting from the alveolar mucosa.

## CASE REPORT

**Figure 1:**
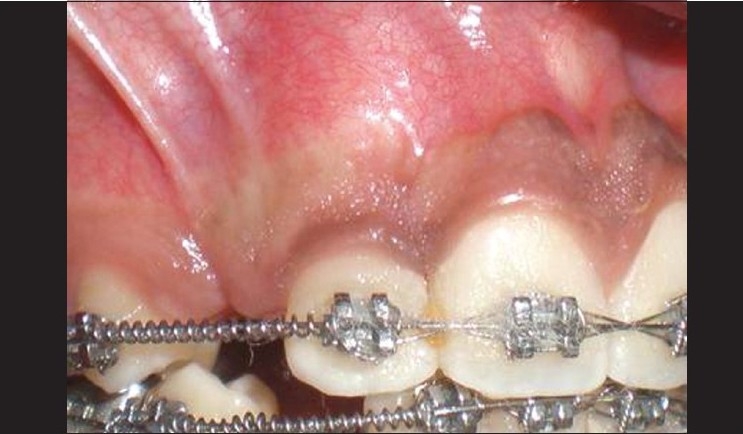
Preoperative view

**Figure 2:**
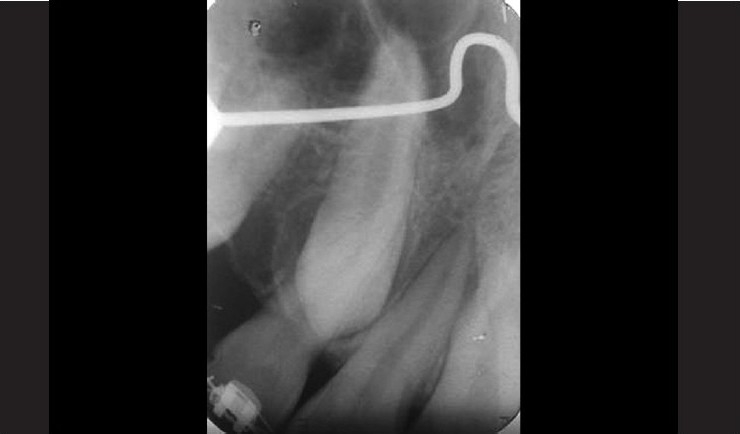
Preoperative X-ray

The patient was a healthy 14 year-old male. A delayed eruption of the maxillary right canine was noted upon intraoral and radiographic examination [Figures 1 and 2]. On palpation, the tooth showed an erupting position that was facial to the crest of the alveolar process and entirely within the alveolar mucosa. Apically positioned partial-thickness flap is the procedure of choice for soft tissue management.

### Procedure [Figures 3–5]

**Figure 3:**
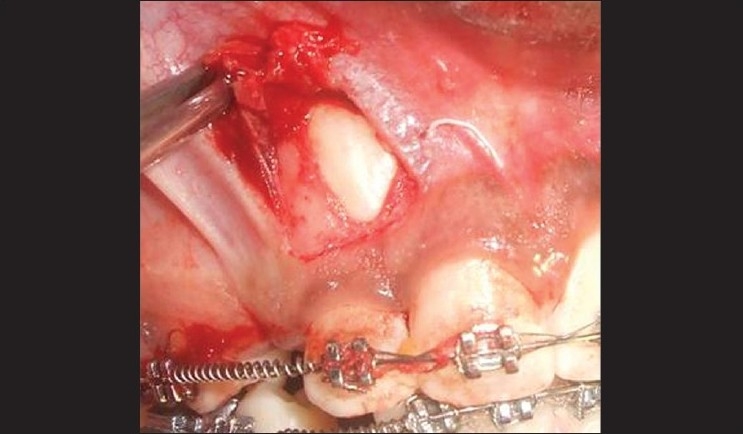
Flap reflected

**Figure 4:**
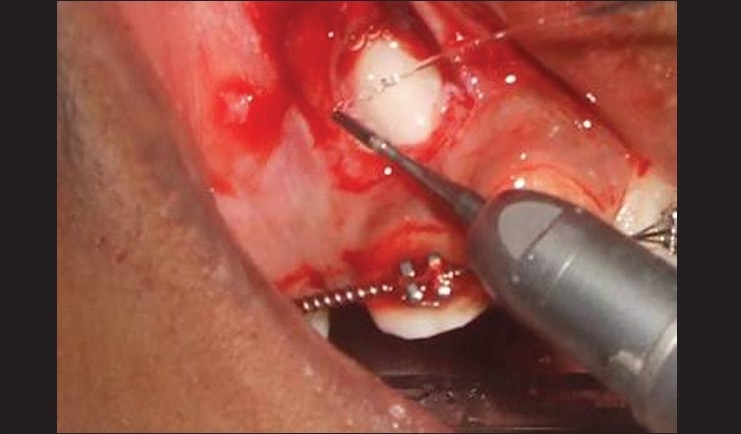
Osteoplasty done around the impacted tooth

**Figure 5:**
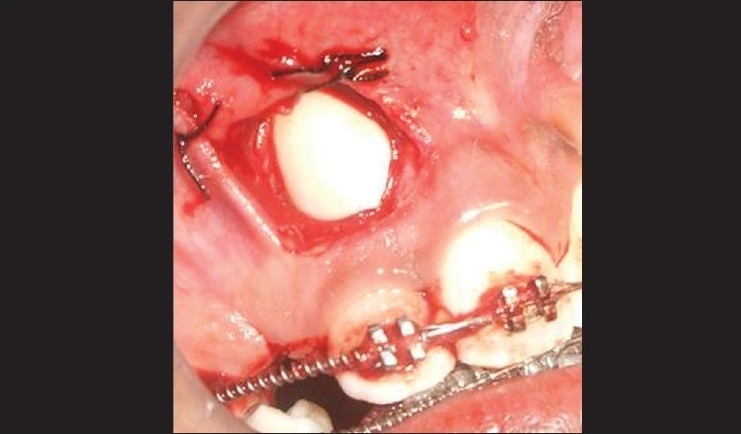
Flap sutured apically

A split-thickness flap was raised by sharp dissection using a number 15 scalpel blade along the tip of the unerupted canine.

1. Vertical releasing incisions were carried high enough into the vestibule to permit apical positioning of the flaps.
2. The flap was wide enough to maintain adequate vascularity.
3. The flap was raised to permit the exposure of impacted tooth. Osteoplasty was done to expose the crown portion for bracket placement.
4. The flap was apically positioned and stabilized with 4-0 silk sutures.

**Figure 6:**
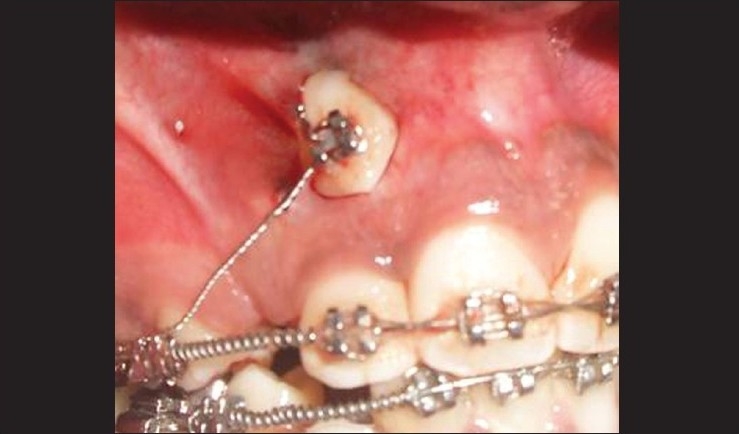
Ten days postoperative view

**Figure 7:**
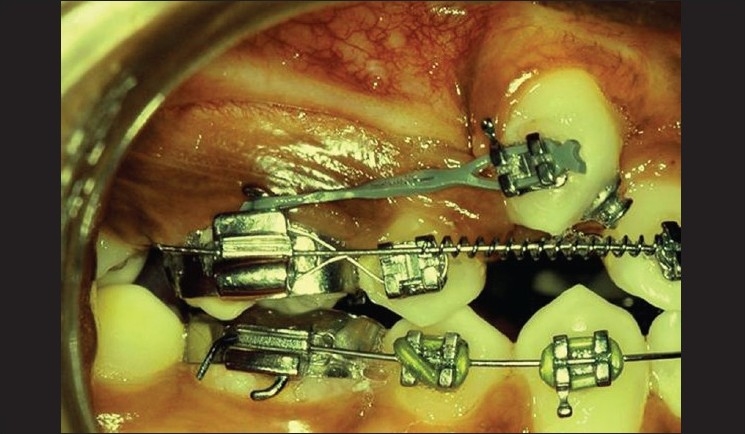
Six months postoperative view

The patient was given oral hygiene instructions that included chlorhexidine rinsing of the mouth for seven days, but no tooth-brushing. One week later, sutures were removed and the area evaluated. Clinical examination revealed a favorable response in the absence of bleeding and inflammation. Mechanical tooth-brushing was reinstated one week after the surgery. The impacted tooth was cleaned and scaled to permit bonding. An orthodontic bracket or button was bonded to position [Figure 6]. After three weeks, the surgical site had revealed an adequate width of keratinized gingiva. The patient was followed up by his orthodontist to bring the maxillary canine into proper occlusion. The six month postoperative view is depicted in Figure 7.

## DISCUSSION

Mucogingival surgery was performed to create a healthy band of keratinized gingiva through careful manipulation of the gingival epithelium. Gingival inflammation may result in gingival recession in areas with no keratinized tissue. Treating or not treating mucogingival problems, before or after recession has occurred, remains controversial. The rationale for performing mucogingival therapy in the child or adolescent varies from treating an adult. For instance, the majority of young patients are not known to practise good oral hygiene which is essential for plaque control. Moreover, pediatric patients present with a greater concern over their root sensitivity and cosmetic appearance. Medico-legal issues should also be addressed in this case as the patient is entitled to be informed about the outcome of not receiving mucogingival therapy that may result in potential gingival complications. All these factors play an important role in determining the decision to perform mucogingival therapy.

A study by Lang and Loe (1972) demonstrated that although tooth surfaces may be kept free of clinically detectable plaque, areas with less than two millimeters of keratinized gingiva tend to remain inflamed. The study proposed that a movable gingival margin would facilitate the introduction of microorganisms into the gingival crevice, resulting in a thin subgingival bacterial plaque that would be difficult to detect and not easily removed by conventional tooth-brushing.[10] The gingival integrity is augmented by creating a band of keratinized gingiva.

During orthodontic treatment, it is particularly important to maintain a healthy band of keratinized gingiva around a labially positioned canine. Otherwise, the mobile tissue around the tooth may strip away from the crown and root surface, leaving a periodontal defect.[11]

A gingivectomy procedure could lead to removal of all the attached gingiva for the canine, and result in an alveolar mucosal attachment. An adequate band of attached gingiva can be achieved by either apically repositioning a flap with attached gingiva, or by grafting keratinized gingiva from the palate to the site of exposure. The present case fulfilled the indications for, namely:

1. Treatment and prevention of mucogingival problems
2. Precise flap stabilization and positioning are required Extensive osseous surgery is not required. Boyd[12] demonstrated that a 2-3 mm band of attached gingival created by an apically repositioned flap in labially positioned canines, is preferable to a window exposure with no attention to keratinized tissue. This technique results in a significant reduction of gingival recession, inflammation, and loss ofattachment.

According to Proffit,[11] there are three categories of problems when dealing with an impacted tooth: Surgical exposure, attachment to the tooth, and orthodontic mechanics to bring the tooth into the arch.

Bishara[13] advocated surgical exposure of the impacted canine with no orthodontic traction only when the tooth has a correct axial inclination. Surgically exposed teeth rarely erupt into a created space without aid, especially once root formation is complete.[14] Removing smaller amounts of overlying bone during surgery results in reduced bone loss after orthodontic treatment. The exposed tooth remained periodontally healthy with proper handling of the soft tissues during the surgical phase and proper postoperative oral hygiene practices. Accordingly, minimal osteoplasty was done in this case to facilitate exposure and eruption of the canine.

Many methods of attaching “hardware” to teeth have been described. Wire ligatures were originally placed around the crown of the impacted tooth, but this had the potential to upset the periodontal attachment.[15]

Boyd[12] compared wire ligation to bonding brackets on impacted canines. In general, wire-ligated teeth had a greater incidence of noneruption, ankylosis, external root resorption, and loss of attached mucosa due to larger flaps required.

With the bracket attached to the tooth after suture removal, the wire already attached to the bracket was then brought up to the arch wire for stabilization and attachment. Six weeks after suture removal, the area had healed uneventfully leaving a band of keratinized gingiva around the canine.

Ideally, mechanical traction should be activated immediately after surgery, and force should be applied to an existing fixed or removable appliance.[16] Traction was activated 7-14 days after the operation. This delay did not produce any long-term orthodontic or periodontal complications.

Other techniques to guide eruption include: Special alignment springs called Ballista springs, coil springs, power chains, or loops of ligature wire extending from the canine to the arch wire.[17]

This case report shows the use of a surgical procedure for uncovering impacted canine, which when used judiciously, gives excellent results and helps in preventing future mucogingivalproblems. Future studies are required to evaluate the long-term efficiency of such procedures.

## CONCLUSION

Although no specific number of millimeters of keratinized gingiva has been proven to be ‘adequate’, clinical judgment factors have been commonly applied that have been useful in assaying the adequacy of keratinized gingiva in individual cases. Nevertheless, the patient in this case presented with potential mucogingival destructive factors that could benefit from a prophylactic mucogingival treatment. The suggested therapy was undertaken to repair the defect before loss of attachment and recession could occur. This treatment demonstrates a more predictable outcome than attempting to cover up any root surface exposure that may develop later. Finally, the importance of allowing the patient to understand the potential defect and its outcome and the options to choose whether or not to receive prophylactic treatment, is clearly the main issue of professional dedication to the patient.

This case report shows that the mucogingival interceptive surgeries, when used judiciously and at appropriate times, can be helpful in preventing future mucogingival problems. This requires a coordinated approach on the part of both the periodontist and the orthodontist, which would ultimately benefit the patient in maintaining a trouble-free periodontium.
